# Paternal occupational exposures and infant congenital heart defects in the Japan Environment and Children’s Study

**DOI:** 10.1265/ehpm.22-00202

**Published:** 2023-02-03

**Authors:** Mina Hayama-Terada, Yuri Aochi, Satoyo Ikehara, Takashi Kimura, Kazumasa Yamagishi, Takuyo Sato, Hiroyasu Iso

**Affiliations:** 1 Osaka Center for Cancer and Cardiovascular Disease Prevention, 1-6-107 Morinomiya, Jyoto-ku, Osaka City, Osaka 536-0025, Japan; 2 Osaka Regional Center for Japan Environment and Children’s Study (JECS), Osaka University, 1-3 Yamadaoka, Suita, Osaka 565-0871, Japan; 3 Division of Environmental Medicine and Population Sciences, Department of Social and Environmental Medicine, Graduate School of Medicine, Osaka University, 1-3 Yamadaoka, Suita, Osaka 565-0871, Japan; 4 Department of Public Health, Hokkaido University Graduate School of Medicine, North 15, West 7, Kita-ku, Sapporo City, Hokkaido 060-8638, Japan; 5 Department of Public Health Medicine, Faculty of Medicine, and Health Service Research Center, University of Tsukuba, 1-1-1 Tennodai, Tsukuba 305-8575, Japan; 6 Department of Maternal and Child Health Information Center, Osaka Women’s and Children’s Hospital, 840 Murodo-cho, Izumi City, Osaka 594-1101, Japan; 7 Institute for Global Health Policy Research, Bureau of International Health Cooperation, National Center for Global Health and Medicine, 1-21-1 Toyama, Shinjuku-ku, Tokyo 162-8655, Japan

**Keywords:** Farther, Occupational exposure, Infant, Congenital heart defects, Cohort study

## Abstract

**Background:**

Few prospective studies have investigated the association between paternal occupational exposures and risk of infant congenital heart defects (CHDs). We investigated the associations between paternal occupational exposures, frequency of use, and concurrent or sequential exposure to a mixture of compounds and the risk of infant CHDs.

**Methods:**

Our study examined 28,866 participants in the Japan Environment and Children’s Study. Logistic regression analysis was used to estimate odds ratios (ORs) and 95% confidence intervals (CIs) associated with paternal occupational exposures during the 3 months until pregnancy was noticed after adjustment for potential confounding factors of the infant CHDs. CHD diagnosis was ascertained from medical record.

**Results:**

In total, 175 were diagnosed with infant CHDs. The number of fathers who were exposed to the following substances at least once a month were: 11,533 for photo copying machine/laser printer, 10,326 for permanent marker, 8,226 for soluble paint/inkjet printer, 6,188 for kerosene/petroleum/benzene/gasoline, 4,173 for organic solvents, 3,433 for chlorine bleach/germicide, 2,962 for engine oil, 2,931 for insecticide, 2,460 for medical sterilizing disinfectant, 1,786 for welding fumes, 1,614 for dyestuffs, 1,247 for any products containing lead-like solder, 986 for herbicide, 919 for radiation/radioactive substances/isotopes, 837 for lead-free solder, 341 for microbes, 319 for formalin/formaldehyde, 301 for agricultural chemical not listed above or unidentified, 196 for general anesthetic for surgery at hospital, 171 for anti-cancer drug, 147 for chromium/arsenic/cadmium, 88 for mercury and 833 for other chemical substances. Paternal occupational exposure regularly to photo copying machine or laser printer and soluble paint/inkjet printer were associated with higher risks of infant CHDs: the adjusted ORs (95%CIs) were 1.38 (1.00–1.91) and 1.60 (1.08–2.37), respectively. The higher risks were also observed for occasional exposure to engine oil, any products containing lead-like solder lead-free solder, and microbes; the adjusted ORs (95%CIs) were 1.68 (1.02–2.77), 2.03 (1.06–3.88), 3.45 (1.85–6.43), and 4.51, (1.63–12.49), respectively.

**Conclusions:**

Periconceptional paternal occupational exposure was associated with a higher risk of infant CHDs. Further studies using biomarkers of the association between paternal occupational exposure and infant CHDs are warranted.

**Supplementary information:**

The online version contains supplementary material available at https://doi.org/10.1265/ehpm.22-00202.

## Introduction

Congenital heart defects (CHDs) are common congenital disorders, and their reported prevalence is the highest in the world, and its etiology includes both genetic and environmental factors [1]. A meta-analysis of 29 case-control and 2 cohort studies about the association between father’s occupation and infant CHDs, published between 1990 and 2018, reported that factory workers, painters janitors, and plywood factory workers had an increased risk of infant CHDs [2]. A prospective and paired case-control study in Italy showed that the presence of paternal occupational exposure was associated with an increased risk of infant CHDs [3]. Another recent case-control study in Hungary reported that paternal occupational exposure to polychlorinated organic substances, phthalates, biphenolic compounds, and solvent was associated with an increased risk of CHDs [4]. In Asia, a case-control study reported that paternal occupational exposure to phthalates and alkylphenolic compound was associated with an increased risk of CHDs [5].

To the best of our knowledge, no large-scale prospective study has simultaneously examined the frequency of use and the combined effects of paternal occupational chemical exposure and the risk of CHD in infants. Risk profiles associated with exposure to common toxic materials have been established, and progressive companies are able to control and manage occupational risks. Moreover, workers can generally reduce their chemical exposure by reducing the frequency of chemical use or by using personal protective devices. However, few studies have evaluated the chronic and sub-chronic effects of chemical mixtures.

The present study aimed to examine the associations between paternal occupational exposures, the frequency of use, and concurrent exposure to each of and a mixture of chemicals during 3 months until partner’s pregnancy was noticed. Our analysis included all fixed data at birth from the Japan Environment and Children’s Study (JECS), a nationwide and government-funded birth cohort study, which was initiated in 2011 [6, 7].

## Methods

### Study design and participants

All data were obtained from the JECS, an ongoing, multicenter, nationwide birth cohort study designed to investigate the environmental factors that affect children’s health and development during the fetal period and/or in early childhood. The study protocol has been described in detail previously [6, 7]. Briefly, pregnant women (mothers) and their partners (fathers) were recruited between January 2011 and March 2014. The study of the participating child’s father continued through March 2014 until the father had completed his one-month checkup after his child’s birth. The eligibility criteria for the participating expectant mothers were as follows: 1) residing in the study area at the time of recruitment; 2) expected delivery date after August 01, 2011; and 3) ability to comprehend the Japanese language and complete the self-administered questionnaire [8]. The present study used the dataset jecs-ag-20160424, which was released in June 2016 and revised in October 2016, along with the supplementary dataset jecs-ag-20160424-sp1. In total, the present investigation consisted of information from 50,577 fathers. We excluded fathers if their partner withdrew from the study (n = 90), information on occupational hazards was missing (n = 7,028), or the medical records did not state the nature of the birth infants (singleton or multiple birth infants) (n = 107), stillbirth/miscarriage (n = 2,464), missing data for father’s age (n = 85), missing data for mother’s age (n = 1) and fathers who answered the questionnaire about their occupation after their child’s birth (n = 12,026). A total of 28,866 fathers were enrolled in the study.

### Outcomes, exposures, and covariates

The primary outcome was the occurrence of CHDs in infants. Information on CHDs diagnoses was ascertained from medical record transcriptions at birth. The physicians, midwives/nurses, and research coordinators made transcripts of maternal and infant medical records. From these, each infant’s sex, birth weight, and congenital anomalies were extracted.

Information on paternal work status, occupation, and occupational exposure during the first trimester of pregnancy was collected using a questionnaire. Each infant’s father answered questions about their main occupation, employment status, and the frequency of occupational exposure. Occupation was based on the Japan Standard Occupational Classification (2009; http://www.soumu.go.jp/english/dgpp_ss/seido/shokgyou/index-co.htm). Fathers were classified according to their type of employment after answering questions related to their primary activity. Four groups were identified: 1) regular employes 2) self-employed workers, defined as those who operated on their own; 3) non-regular employes, including part-time or dispatched workers from temporary labor agencies; and 4) unemployed and others, including house persons or persons who were on a leave of absence. Non-regular employed workers were defined according to the Act for Securing the Proper Operation of Worker Dispatching Undertakings and Improved Working Conditions for Dispatched Workers (Act No. 88 of 1985, hereinafter referred to as Worker Dispatching Law). Participants were asked how many times they used the following 23 chemicals spent more than half a day on the working, during the 3 months until their partner’s pregnancy was noticed. They were given the answer choices “no, 1–3 times a month, 1–6 times a week or everyday” for each chemical. The paternal occupational exposure included: 1) photo copying machine/laser printer, 2) permanent marker, 3) soluble paint/inkjet printer, 4) kerosene/petroleum/benzene/gasoline, 5) organic solvents, 6) chlorine bleach/germicide, 7) engine oil, 8) insecticide, 9) medical sterilizing disinfectant, 10) welding fumes, 11) dyestuffs (hair coloring), 12) any products containing lead-like solder, 13) herbicide, 14) radiation/radioactive substances/isotopes, 15) lead-free solder, 16) microbes, 17) formalin/formaldehyde, 18) agricultural chemical not listed above or unidentified, 19) general anesthetic for surgery at hospital, 20) anti-cancer drug (not including your own remedy), 21) chromium/arsenic/cadmium, 22) mercury, 23) other chemical substances. Exposure was defined as occurring for more than one-half of a day at work, and the frequencies were categorized into three groups (no, occasional and regular) for 3 months until the pregnancy was noticed. Occasional exposure was defined as exposure at 1–3 times a month, and regular exposure was defined as exposure at 1–6 times a week or every day.

The potential confounders measured in the JECS were following: father’s and mother’s age (quintiles), self-reported medical history of CHDs (yes or no), educational attainment (high school or less, vocational school, junior college, more than university or higher), smoking habits during pregnancy (never smoking, quit before pregnancy, quit after becoming pregnant, or smoking during pregnancy), drinking habits during pregnancy (never drinking, quit before pregnancy, quit after becoming pregnant, or drinking during pregnancy), father’s history of diabets mellitus (yes or no), mother’s histories of gestational diabets mellitus, epilepsy, connective tissue disease, rubella infection, periconceptional drug exposure to lithium carbonate, amphetamine, or hydantoin which are known to be teratogenic (yes or no), father’s body mass index and mother’s body mass index before pregnancy, and household income (<2, 2–3.9, 4–5.9, 6–7.9, or ≥8 million yen/year).

### Statistical analysis

The characteristics of fathers, mothers, and children were compared between fathers who had occupational exposure and those who did not. The differences between the mean values of continuous variables and proportions of dichotomous variables were tested using Student’s *t*-test and Chi-squared analysis, respectively. Logistic regression analysis was used to estimate the odds ratios (ORs) and 95% confidence intervals (CIs) for the association between paternal occupational exposure and the risk of infant CHDs. The use of the paternal occupational exposure was classified as either occasional or regular, after which, it was categorized in greater detail as follows: no exposure, occasional only, occasional with other exposures, regular only, regular with other exposures. The ORs were adjusted for potential confounding factors stated above. All statistical analyses were performed using SAS software (version 9.4; SAS Institute, Cary, NC, USA), and a two-tailed p-value < 0.05 was considered statistically significant.

## Results

Participant’s characteristics according to the presence or absence of the fathers’ occupational exposure at least once a month (occasionally or regularly) are shown in Table 1. Fathers who were exposed at least once a month (occasionally or regularly) were younger and were more likely to be smokers during pregnancy than those who were not exposed. There was no difference in the proportion of fathers with a history of CHDs between the two groups. Regarding the job categories, exposed fathers were more often self-employed than non-exposed fathers. Exposed fathers were more likely to be agriculture, forestry or fishery workers, manufacturing process workers, construction and mining workers, workers not classified by occupation and less likely to be administrative and managerial workers, professional and engineering workers, clerical workers, sales workers, security workers, transport and machine operation workers, or those engaged in carrying, cleaning, packaging, and related jobs. The prevalence of occupational exposure to photo copying machines and laser printers was the highest (67.0%), followed by permanent markers (60.0%), soluble paint/inkjet printer (47.8%), kerosene/petroleum/benzene/gasoline (35.9%), organic solvents (24.2%) and so on. The proportion of infants with CHDs was higher among exposed fathers than among unexposed fathers.

**Table 1:** Characteristics of children’s fathers and children according to the father’s occupational exposure.

**Variables**	**Father’s occupational exposure**			
	**Yes** **(n = 17,219)**		**No** **(n = 11,647)**	
**Fathers**				
Age (years)	32 ± 5.8		33 ± 5.9	
Current smoking	7,683	44.6%	4,556	39.1%
Current drinking	12,875	74.8%	8,726	74.9%
History of CHDs	45	0.3%	24	0.2%
Job categories				
Regular employes	14,316	83.1%	9,755	83.8%
Self-employed workers	1,461	8.5%	850	7.3%
Non-regular employes	829	4.8%	574	4.9%
Unemployed and others	179	1.0%	145	1.2%
Occupational classification				
Administrative and managerial workers	591	3.4%	543	4.7%
Professional and engineering workers	5,248	30.5%	3,267	28.1%
Clerical workers	1,422	8.3%	1,268	10.9%
Sales workers	1,697	9.9%	1,399	12.0%
Service workers	1,892	11.0%	1,321	11.3%
Security workers	717	4.2%	559	4.8%
Agriculture, forestry and fishery workers	325	1.9%	140	1.2%
Manufacturing process workers	2,447	14.2%	1,145	9.8%
Transport and machine operation workers	535	3.1%	586	5.0%
Construction and mining workers	1,087	6.3%	547	4.7%
Carrying, cleaning, packaging, and related jobs	210	1.2%	174	1.5%
Workers not classified by occupation	394	2.3%	211	1.8%
Compounds of occupational exposures				
Photo copying machine/laser printer	11,533	67.0%	—	—
Permanent marker	10,326	60.0%	—	—
Soluble paint/inkjet printer	8,226	47.8%	—	—
Kerosene/petroleum/benzene/gasoline	6,188	35.9%	—	—
Organic solvents	4,173	24.2%	—	—
Chlorine bleach/germicide	3,433	19.9%	—	—
Engine oil	2,962	17.2%	—	—
Insecticide	2,931	17.0%	—	—
Medical sterilizing disinfectant	2,460	14.3%	—	—
Welding fumes	1,786	10.4%	—	—
Dyestuffs (hair coloring)	1,614	9.4%	—	—
Any products containing lead-like solder	1,247	7.2%	—	—
Herbicide	986	5.7%	—	—
Radiation/radioactive substances/isotopes	919	5.3%	—	—
Lead-free solder	837	4.9%	—	—
Microbes	341	2.0%	—	—
Formalin/formaldehyde	319	1.9%	—	—
Agricultural chemical not listed above or unidentified	301	1.7%	—	—
General anesthetic for surgery at hospital	196	1.1%	—	—
Anti-cancer drug (not including your own remedy)	171	1.0%	—	—
Chromium/arsenic/cadmium	147	0.9%	—	—
Mercury	88	0.5%	—	—
Other chemical substances	833	4.8%	—	—

**Children**				
Male gender	8,796	51.1%	5,879	50.5%
Birth weight (g)	3,018 ± 416.0		3,025 ± 412.5	
CHDs	120	0.7%	55	0.5%

Data are expressed as mean ± SD, or Number (%).

Mother’s characteristics according to the father’s occupational exposure are shown in Supplemental Table 1. Again, there were no difference in the proportion of mothers with a history of CHDs. The prevalence of occupational exposures was lower in mothers compared with fathers exposed for chlorine bleach/germicide (18.6%) and anti-cancer drug (1.3%) with similar exposure in fathers.

Among 28,866 live births, 175 infants were diagnosed with CHDs (6.06/1,000 live births). Table 2 presents the associations between periconceptional paternal occupational exposures and the risk of infant CHDs. Paternal occupational exposure regularly to photo copying machine/laser printer and soluble paint/inkjet printer were associated with higher risks of infant CHDs: the adjusted ORs (95%CIs) were 1.38 (1.00–1.91) and 1.60 (1.08–2.37), respectively. The higher risks were also observed for parental exposure occasionally to engine oil, any products containing lead-like solder, lead-free solder, and microbes; the adjusted ORs (95%CIs) were 1.68 (1.02–2.77), 2.03 (1.06–3.88), 3.45 (1.85–6.43), and 4.51 (1.63–12.49), respectively.

**Table 2:** Associations between paternal occupational exposures and the risk of infant congenital heart defects

**Occupational exposures**		**Infant congenital heart defects**		

	**Number at ** **risk**	**Number of ** **cases**	**OR (95%CI)**	
			**Unadjusted**	**Adjusted**
**Photo copying machine/laser printer**				
No	17,333	95	(Reference)	(Reference)
Occasional	2,966	16	0.98 (0.58–1.67)	1.00 (0.59–1.71)
Regular	8,567	64	1.37 (0.99–1.88)	1.38 (1.00–1.91)
**Permanent marker**				
No	18,540	106	(Reference)	(Reference)
Occasional	5,346	34	0.98 (0.58–1.67)	1.14 (0.77–1.68)
Regular	4,980	35	1.37 (0.99–1.88)	1.30 (0.88–1.92)
**Soluble paint/inkjet printer**				
No	20,640	113	(Reference)	(Reference)
Occasional	4,292	29	1.24 (0.82–1.86)	1.22 (0.81–1.84)
Regular	3,934	33	1.54 (1.04–2.27)	1.60 (1.08–2.37)
**Kerosene/petroleum/benzene/gasoline**				
No	22,678	143	(Reference)	(Reference)
Occasional	3,902	17	0.69 (0.42–1.14)	0.72 (0.43–1.19)
Regular	2,286	15	1.04 (0.61–1.78)	1.10 (0.64–1.90)
**Organic solvents**				
No	24,693	145	(Reference)	(Reference)
Occasional	2,113	19	1.54 (0.95–2.48)	1.57 (0.96–2.55)
Regular	2,060	11	0.91 (0.49–1.68)	0.95 (0.51–1.77)
**Chlorine bleach/germicide**				
No	25,433	150	(Reference)	(Reference)
Occasional	2,444	22	1.53 (0.98–2.40)	1.54 (0.98–2.42)
Regular	989	3	0.51 (0.16–1.61)	0.54 (0.17–1.71)
**Engine oil**				
No	25,904	155	(Reference)	(Reference)
Occasional	1,861	18	1.62 (0.99–2.65)	1.68 (1.02–2.77)
Regular	1,101	2	0.30 (0.08–1.22)	0.34 (0.08–1.37)
**Insecticide**				
No	25,935	151	(Reference)	(Reference)
Occasional	2,551	20	1.35 (0.85–2.16)	1.36 (0.85–2.17)
Regular	380	4	1.82 (0.67–4.93)	1.84 (0.67–5.03)
**Medical sterilizing disinfectant**				
No	26,406	159	(Reference)	(Reference)
Occasional	1,006	8	1.32 (0.65–2.70)	1.34 (0.65–2.74)
Regular	1,454	8	0.91 (0.45–1.86)	1.01 (0.49–2.08)
**Welding fumes**				
No	27,080	166	(Reference)	(Reference)
Occasional	1,073	7	1.07 (0.50–2.27)	1.07 (0.50–2.31)
Regular	713	2	0.46 (0.11–1.84)	0.48 (0.12–1.95)
**Dyestuffs (hair coloring)**				
No	27,252	161	(Reference)	(Reference)
Occasional	1,399	13	1.58 (0.90–2.78)	1.58 (0.89–2.81)
Regular	215	1	—	—
**Any products containing lead-like solder**				
No	27,619	162	(Reference)	(Reference)
Occasional	867	10	1.98 (1.04–3.76)	2.03 (1.06–3.88)
Regular	380	3	1.35 (0.43–4.25)	1.44 (0.46–4.57)
**Herbicide**				
No	27,880	171	(Reference)	(Reference)
Occasional	915	4	0.71 (0.26–1.92)	0.70 (0.26–1.90)
Regular	71	0	—	—
**Radiation/radioactive substances/isotopes**				
No	27,947	169	(Reference)	(Reference)
Occasional	398	4	1.67 (0.62–4.52)	1.61 (0.59–4.42)
Regular	521	2	0.63 (0.16–2.56)	0.64 (0.16–2.58)
**Lead-free solder**				
No	28,029	164	(Reference)	(Reference)
Occasional	580	11	3.29 (1.77–6.08)	3.45 (1.85–6.43)
Regular	257	0	—	—
**Microbes**				
No	28,525	170	(Reference)	(Reference)
Occasional	145	4	4.74 (1.73–12.94)	4.51 (1.63–12.49)
Regular	196	1	—	—
**Formalin/formaldehyde**				
No	28,547	173	(Reference)	(Reference)
Occasional	202	2	1.64 (0.40–6.66)	1.41 (0.34–5.79)
Regular	117	0	—	—
**Agricultural chemical not listed above or unidentified**				
No	28,565	173	(Reference)	(Reference)
Occasional	233	2	1.42 (0.35–5.76)	1.49 (0.36–6.08)
Regular	68	0	—	—
**General anesthetic for surgery at hospital**				
No	28,670	173	(Reference)	(Reference)
Occasional	93	2	3.62 (0.89–14.81)	3.70 (0.89–15.37)
Regular	103	0	—	—
**Anti-cancer drug (not including your own remedy)**				
No	28,695	174	(Reference)	(Reference)
Occasional	112	1	—	—
Regular	59	0	—	—
**Chromium/arsenic/cadmium**				
No	28,719	174	(Reference)	(Reference)
Occasional	84	1	—	—
Regular	63	0	—	—
**Mercury**				
No	28,778	175	(Reference)	(Reference)
Occasional	69	0	—	—
Regular	19	0	—	—
**Other chemical substances**				
No	28,033	167	(Reference)	(Reference)
Occasional	294	2	1.14 (0.28–4.63)	1.02 (0.25–4.17)
Regular	539	6	1.88 (0.83–4.26)	1.87 (0.82–4.25)

Adjusted for age, histories of CHDs and diabetes mellitus, body mass index, educational attainment, household income, smoking status, and drinking status of father and mother, as well as epilepsy, connective tissue disease, rubella, periconceptional drug exposure of mothers.

Table 3 presents the associations between combined paternal occupational exposures and the risk of infant CHDs. Paternal exposure regularly to photo copying machine/laser printer, and soluble paint/inkjet printer with other exposures were associated with higher risks of infant CHDs; the adjusted ORs (95%CIs) were 1.47 (1.06–2.05) and 1.61 (1.09–2.39), respectively. The higher risks were also observed for paternal exposures occasionally to organic solvents, chlorine bleach/germicide, engine oil, any products containing lead-like solder, lead-free solder, and microbes with other exposures; the adjusted ORs (95%CIs): 1.69 (1.04–2.74), 1.57 (1.00–2.46), 1.69 (1.01–2.81), 2.07 (1.08–3.96), 3.29 (1.72–6.31), and 3.71 (1.16–11.90). respectively.

**Table 3:** Associations between paternal combined occupational exposures and the risk of infant congenital heart defects.

**Occupational exposures**		**Infant congenital heart defects**		

	**Number at ** **risk**	**Number of ** **cases**	**OR (95%CI)**	
			**Unadjusted**	**Adjusted**
**Photo copying machine/laser printer**				
No	17,333	95	(Reference)	(Reference)
Occasional only	358	1	—	—
Occasional with other exposures	2,608	15	1.05 (0.61–1.81)	1.07 (0.62–1.85)
Regular only	1,243	6	0.88 (0.39–2.01)	0.84 (0.37–1.94)
Regular with other exposures	7,324	58	1.45 (1.04–2.01)	1.47 (1.06–2.05)
**Soluble paint/inkjet printer**				
No	20,640	113	(Reference)	(Reference)
Occasional only	55	0	—	—
Occasional with other exposures	4,237	29	1.25 (0.83–1.89)	1.24 (0.82–1.87)
Regular only	26	0	—	—
Regular with other exposures	3,908	33	1.55 (1.05–2.28)	1.61 (1.09–2.39)
**Organic solvents**				
No	24,693	145	(Reference)	(Reference)
Occasional only	149	0	—	—
Occasional with other exposures	1,964	19	1.65 (1.02–2.67)	1.69 (1.04–2.74)
Regular only	191	1	—	—
Regular with other exposures	1,869	10	0.91 (0.48–1.73)	0.95 (0.50–1.82)
**Chlorine bleach/germicide**				
No	25,433	150	(Reference)	(Reference)
Occasional only	47	0	—	—
Occasional with other exposures	2,397	22	1.56 (1.00–2.45)	1.57 (1.00–2.46)
Regular only	37	0	—	—
Regular with other exposures	952	3	0.53 (0.17–1.67)	0.57 (0.18–1.79)
**Engine oil**				
No	25,904	155	(Reference)	(Reference)
Occasional only	102	1	—	—
Occasional with other exposures	1,759	17	1.62 (0.98–2.68)	1.69 (1.01–2.81)
Regular only	90	0	—	—
Regular with other exposures	1,011	2	0.33 (0.08–1.33)	0.36 (0.09–1.48)
**Any products containing lead-like solder**				
No	27,619	162	(Reference)	(Reference)
Occasional only	17	0	—	—
Occasional with other exposures	850	10	2.02 (1.06–3.84)	2.07 (1.08–3.96)
Regular only	13	1	—	—
Regular with other exposures	367	2	0.93 (0.23–3.76)	0.99 (0.24–4.04)
**Lead-free solder**				
No	28,029	164	(Reference)	(Reference)
Occasional only	24	1	—	—
Occasional with other exposures	556	10	3.11 (1.63–5.93)	3.29 (1.72–6.31)
Regular only	13	0	—	—
Regular with other exposures	244	0	—	—
**Microbes**				
No	28,525	170	(Reference)	(Reference)
Occasional only	9	1	—	—
Occasional with other exposures	136	3	3.76 (1.19–11.93)	3.71 (1.16–11.90)
Regular only	19	1	—	—
Regular with other exposures	177	0	—	—

Adjusted for age, histories of CHDs and diabetes mellitus, body mass index, educational attainment, household income, smoking status, and drinking status of father and mother, as well as epilepsy, connective tissue disease, rubella, periconceptional drug exposures of mothers.

## Discussion

This nationwide cohort study performed a broad examination of the associations between paternal periconceptional occupational exposure to 23 substances and the risk of infant CHDs.

The higher risks of infant CHDs were observed for the regular exposure to photo copying machine/laser printer, soluble paint/inkjet printer and engine oil, and the occasional (but not regular) exposure to engine oil, any products containing lead-like solder, lead-free solder, and microbes. The findings were similar when combined with other exposures except for occasional exposure to organic solvents and chlorine bleach/germicides which appeared as associated factors for the excess risk. One of the probable reasons for the occasional (but not regular) exposure to several chemicals and microbes was the smaller number of people at regular exposure and the limited cases of infant CHDs probably due to the strict regulation for these chemicals to minimize the workers’ exposure. In our study, maternal exposure to any of these was not associated with the risk of infant CHDs although organochlorine chemicals, widely used as insecticide, herbicide and other agricultural chemicals, reported to disrupt oocyte maturation and follicle physiology in animal studies [9].

Potential mechanisms for explaining our findings are as follows. First, as for photo copying machine/laser printer, no observational studies have reported the association with risk of infant CHDs, and no animal studies have investigated the mechanism. Our finding could be due to other combined exposures or observed by chance. Second, as for soluble paint/inkjet printer, the parental exposure to organic solvents, include in soluble paint/inkjet printer, was associated with a two-fold increased risk of infant CHDs in a recent cross-sectional study [10]. In-vitro study showed that the hexane fraction of ethanol substance, one of organic solvents, reduced sperm motility and viability probably due to disruption of lipid within the sperm membrane [11]. Third, as for engine oil, the exposure to used engine oil, containing heavy metals and polycyclic aromatic induced weight loss, lowered the number of sperm, and increased sperm head deformity according to an animal study using Wister rats [12]. Forth, as for any products containing lead-like solder, lead-free solder, and microbes, no observational studies have reported the association with risk of infant CHDs. Our finding could be due to other combined exposures or observed by chance. However, an animal study using Wister rats showed that bismuth subnitrate, one of the ingredients of lead-free solder, accumulated in Leydig cells and lowered serum testosterone levels, suggesting its toxicity to testicular function [13].

Previous cross-sectional studies investigated the associations of occupation or occupational exposure with the specific types of CHDs. Father’s occupations as production craftsmen/related workers and cleaners/labors/related workers were associated with a higher risk of non-chromosomal single bith defect including CHDs: the adjusted risk ratio (95%CI) = 1.42 (1.10–1.82) and 1.43 (1.07–1.91), respectively [14]. The paternal exposure to soldering with lead/smelting was associated with a single ventricle with normal organ situs: the OR (95%CI) = 2.6 (1.0–6.5) [15].

A case-control study using Bayesian regression analysis showed that father’s work as landscapers and groundskeepers (who may likely be exposed to microbes) was associated with a higher risk of total anomalous pulmonary venous return: the adjusted OR (95%CI) = 1.8 (1.2–2.8) for total anomalous pulmonary venous return and 1.3 (1.0–1.7) for arterial septal defect [16].

Assessing the risk of combined and mixed exposures is a global issue; the US Environmental Protection Agency published guidelines on the risk assessments of chemical mixtures [17, 18], and the Council of the European Union emphasized on the need to consider combined and mixed exposures to chemicals in future risk assessments [19, 20]. Fathers can be exposed to not only a single compound, but also a range of occupational chemicals. However, there is a lack of knowledge regarding the effects of complex exposure. This is the first epidemiological study to evaluate the association between combined paternal occupational exposure and the risk of infant CHDs.

Our study has several limitations. First, we used a self-reported questionnaire to measure the frequency of parental occupational exposures and other covariates, but not the amount of occupational exposures. The frequency of exposures assumed to be misclassified non-differentially, so that the estimated associations were likely to be undermined, making real associations stronger. Second, the biomarkers of occupational exposure were not investigated, leaving the need of future studies. Third, our study could not perform the analysis for type-specific CHDs because of a small number of cases. A previous case-control study investigated the associations between paternal occupational hazards and some types of CHDs; the exposure to polychlorinated compounds was associated with atrioventricular septal defects: the adjusted OR (95%CI) = 4.22 (1.23–14.42), the exposure to phthalates was associated with a perimembranous ventricular septal defect: the adjusted OR (95%CI) = 2.84 (1.37–5.92), and the exposure to alkylphenolic compounds was associated with coarctation of aorta: the adjusted OR (95%CI) = 3.85 (1.17–12.67) [21]. In addition, despite the large sample size of our study, cases of CHDs were sparsely distributed because the classification of paternal occupational exposures yielded many combinations, in which there were no or only a few infants in each exposure group. Multiple statistical testing may cause a false positive finding.

Despite these limitations, no large-scale prospective study has examined the effects of paternal occupational exposure during pregnancy. This study had a strength of a prospective cohort design, unbiased exposure, and outcome estimation, and providing supportive evidence for the management of paternal occupational exposure to substances that might cause infant CHDs.

## Conclusions

The periconceptional paternal occupational exposure was associated with a higher risk of infant CHDs, suggesting the importance of the management of paternal occupational exposures for the prevention of infant CHDs. Further studies using biomarkers of occupational exposure are warranted.

## Abbreviations

CHD: congenital heart defects
JECS: Japan Environment and Children’s Study
OR: odd ratio
CI: confidence interval
DM: diabetes mellitus
GDM: gestational diabetes mellitus

## Supplementary information

**Additional file 1:** **Supplemental Table 1.** Characteristics of children’s mothers according to the father’s occupational exposure.

## Declarations

### Availability of data and material

Data are unsuitable for public deposition due to ethical restrictions and legal framework of Japan. It is prohibited by the Act on the Protection of Personal Information (Act No. 57 of 30 May 2003, amendment on 9 September 2015) to publicly deposit the data containing personal information. Ethical Guidelines for Medical and Health Research Involving Human Subjects enforced by the Japan Ministry of Education, Culture, Sports, Science and Technology and the Ministry of Health, Labour and Welfare also restricts the open sharing of the epidemiologic data. All inquiries about access to data should be sent to: jecs-en@nies.go.jp. The person responsible for handling enquiries sent to this e-mail address is Dr Shoji F. Nakayama, JECS Programme Office, National Institute for Environmental Studies.
