# Supplementary material for: Paternal occupational exposures and infant congenital heart defects in the Japan Environment and Children’s Study
Source: Environ Health Prev Med. 2023 Feb 3;28:12. doi: 10.1265/ehpm.22-00202 (PMC9922566; doi:10.1265/ehpm.22-00202)
Supplement: Supplementary file 1 — Additional file 1: Supplemental Table 1. Characteristics of children’s mothers according to the father’s occupational exposure. [file ehpm-28-012-s001.pdf]

Supplemental Table 1. Characteristics of children's mothers according to the father's occupational exposure.

| Variables | Father's occupational exposure | | | | | |
|---|---|---|---|---|---|---|
| | Yes (n=17,219) | | | No (n=11,647) | | |
| Age (years) | 30 | ± | 5.0 | 31 | ± | 4.8 |
| Current smoking | 710 | | 4.1% | 365 | | 3.1% |
| Current drinking | 450 | | 2.6% | 280 | | 2.4% |
| Use of drugs | 1,907 | | 11.1% | 1,204 | | 10.3% |
| History of CHDs | 52 | | 0.3% | 43 | | 0.4% |
| DM/GDM | 143 | | 0.8% | 90 | | 0.8% |
| Rubella | 296 | | 1.7% | 204 | | 1.8% |
| Connective tissue disease | 22 | | 0.1% | 17 | | 0.1% |
| Epilepsy | 108 | | 0.6% | 62 | | 0.5% |
| Compounds of occupational exposures | | | | | | |
| Photo copying machine/laser printer | 4,802 | | 27.9% | — | | — |
| Permanent marker | 5,001 | | 29.0% | — | | — |
| Soluble paint/inkjet printer | 3,327 | | 19.3% | — | | — |
| Kerosene/petroleum/benzene/gasoline | 1,629 | | 9.5% | — | | — |
| Organic solvents | 149 | | 0.9% | — | | — |
| Chlorine bleach/germicide | 3,197 | | 18.6% | — | | — |
| Engine oil | 102 | | 0.6% | — | | — |
| Insecticide | 982 | | 5.7% | — | | — |
| Medical sterilizing disinfectant | 1,998 | | 11.6% | — | | — |
| Dyestuffs (hair coloring) | 1,114 | | 6.5% | — | | — |
| Any products containing lead-like solder | 61 | | 0.4% | — | | — |
| Herbicide | 106 | | 0.6% | — | | — |
| Radiation/radioactive substances/isotopes | 372 | | 2.2% | — | | — |
| Lead-free solder | 24 | | 0.1% | — | | — |
| Microbes | 92 | | 0.5% | — | | — |
| Formalin/formaldehyde | 136 | | 0.8% | — | | — |
| Agricultural chemical not listed above or unidentified | 28 | | 0.2% | — | | — |
| General anesthetic for surgery at hospital | 160 | | 0.9% | — | | — |
| Anti-cancer drug (not including your own remedy) | 219 | | 1.3% | — | | — |
| Chromium/arsenic/cadmium | 9 | | 0.1% | — | | — |
| Mercury | 58 | | 0.3% | — | | — |
| Other chemical substances | 156 | | 0.9% | — | | — |
